# Childhood Socioeconomic Disadvantage and Pathways to Memory Performance in Mid to Late Adulthood: What Matters Most?

**DOI:** 10.1093/geronb/gbac075

**Published:** 2022-05-18

**Authors:** Katherine J Ford, Lindsay C Kobayashi, Anja K Leist

**Affiliations:** Institute for Research on Socio-Economic Inequality (IRSEI), University of Luxembourg, Esch-sur-Alzette, Luxembourg; Department of Epidemiology, University of Michigan School of Public Health, Ann Arbor, Michigan, USA; Institute for Research on Socio-Economic Inequality (IRSEI), University of Luxembourg, Esch-sur-Alzette, Luxembourg

**Keywords:** Cognition, Early-life conditions, Education, Mediation, Occupation

## Abstract

**Objectives:**

Childhood socioeconomic disadvantage is consistently associated with lower cognitive function in later life. This study aims to distinguish the contribution of specific aspects of childhood socioeconomic disadvantage for memory performance in mid to late adulthood, with consideration for direct and indirect effects through education and occupation.

**Methods:**

Data were from adults aged 50 to 80 years who completed the life history module in the 2006/2007 wave of the English Longitudinal Study of Aging (*n* = 4,553). The outcome, memory score, was based on word recall tests (range: 0–20 points). We used the g-formula to estimate direct and indirect effects of a composite variable for childhood socioeconomic disadvantage and its 4 individual components: lower-skilled occupation of the primary breadwinner, having few books in the home, overcrowding in the home, and lack of water and heating facilities in the home.

**Results:**

Few books were the most consequential component of childhood socioeconomic disadvantage for later-life memory (total effect: −0.82 points for few books; 95% confidence interval [CI]: −1.04, −0.60), with roughly half being a direct effect. The total effect of a breadwinner in lower-skilled occupations was smaller but not significantly different from a few books (−0.67 points; 95% CI: −0.88, −0.46), while it was significantly smaller with overcrowding (−0.31 points; 95% CI: −0.56, −0.06). The latter 2 total effects were mostly mediated by education and occupation.

**Discussion:**

A literate environment in the childhood home may have lasting direct effects on memory function in mid to later life, while parental occupation and overcrowding appear to influence memory primarily through educational and occupational pathways.

The investigation of early childhood conditions is fundamental to improving our understanding of the effects of cumulative socioeconomic disadvantage over the life course on later-life cognitive function. However, this is a challenging research area due to the multifaceted nature of socioeconomic position in childhood and the numerous potential life course pathways to consider. Cognitive reserve theory suggests that lifetime engagement in cognitively stimulating activities protect against the deleterious effects of aging-related brain pathologies on cognitive function (Stern, 2012). On the other hand, the cognitive gains from intellectual activities may plateau in late adolescence (Kremen et al., 2019). Childhood and adolescence may then represent a sensitive life course period for the consolidation of cognitive health (Ben-Shlomo & Kuh, 2002).

Childhood socioeconomic disadvantage could affect late-life cognition through its influence on early-life neurodevelopment (Jensen et al., 2017), or through influencing unequal educational and occupational opportunities (Ford & Leist, 2021; Leist et al., 2021). Neurodevelopmental pathways could be understood as the physiological embodiment of the early social environment (Krieger, 2005). Longer durations of disadvantage in childhood are associated with poorer memory performance in adolescence, with this relationship being mediated through physiological indicators of chronic stress (Evans & Schamberg, 2009). Similarly, adult height, which is thought to be a stable biomarker of nutrition during critical growth periods in childhood, is correlated with subsequent educational attainment, employment, earnings, and cognitive abilities in adulthood (Case & Paxson, 2010; Perkins et al., 2016).

Early-life socioeconomic disadvantage may limit educational attainment through influencing early childcare options and access to quality schooling (Fort et al., 2019; Ruzek et al., 2014; van de Werfhorst, 2021). Throughout childhood, economic adversity is associated with fewer parent–child conversations, less integration in the school community, fewer books, and more television watching in the home (Evans, 2004), all of which may have consequences on academic success. Moreover, even when similar levels of education are attained, occupational outcomes may differ by family origins. Explanations could include differences in institutional prestige of schools, cultural capital, and social networks, which may influence occupational opportunities following education (Abrahams, 2017; Rivera, 2011). Despite extensive increases in white-collar jobs over the mid-Twentieth century, those with working-class parents are most likely to be working-class themselves, a pattern which is repeated moving up the occupational class ladder (Goldthorpe & Payne, 1986). The lack of both educational and occupational opportunities has seemingly important consequences on cognitive performance in later adulthood (Ford & Leist, 2021; Leist et al., 2021).

## Previous Research

To date, many studies have explored the direct effects of composite childhood socioeconomic status (cSES) indicators on adult cognition alongside indirect effects through adult educational and occupational attainment and other health factors (Aartsen et al., 2019; Beck et al., 2018; Cermakova et al., 2018; Greenfield & Moorman, 2019; Lyu & Burr, 2016; McElroy et al., 2021; Oi & Haas, 2019; Wolfova et al., 2021). Whether this relationship is fully or partially mediated by adult socioeconomic characteristics is debatable, with some finding full mediation (Beck et al., 2018; McElroy et al., 2021; Oi & Haas, 2019); and others partial mediation (Aartsen et al., 2019; Cermakova et al., 2018; Wolfova et al., 2021). The majority of these studies have investigated these relationships with structural equation modeling (SEM) or traditional (Baron and Kenny) mediation than with approaches from the potential-outcomes framework, such as the g-formula. Traditional mediation methods can rarely meet all confounder assumptions needed for unbiased estimates, while SEM approaches are tied to models using linear, normally-distributed variables with subsequent interpretational challenges (VanderWeele, 2016).

## Composite Measures of Childhood Disadvantage

The use of one indicator of socioeconomic position will not capture the entirety of the disadvantaged experience (Galobardes et al., 2006a). However, composite measures constructed from a set of observed values are subject to interpretational confounding, where the broad understanding of a concept is attributed to composite measures across different studies despite them being empirically different (Howell et al., 2007). In this case, the concept of childhood socioeconomic disadvantage in one setting may mean something different in another depending on relationships between the underlying observable components and outcomes in each context (Howell et al., 2007).

Different indicators of cSES represent different aspects of disadvantage (Galobardes et al., 2006a). Housing conditions, like overcrowding or lack of heating, may reflect increased exposure to stress, infections, and competing needs such as food (Evans, 2004; Galobardes et al., 2006a), with important connections to general neurodevelopment for young people (Case & Paxson, 2010; Jensen et al., 2017). Likewise, parental occupation could reflect household income and subsequent material standards of living (Galobardes et al., 2006a). Yet parental occupation may also approximate the intellectual environment of a household unit (Galobardes et al., 2006a). Parental occupation can shape occupational ambitions for children early on, with schools seeming to have comparatively little influence (Pimlott-Wilson, 2011). The number of books in the childhood home is another indicator of cultural capital and social advantage (Sieben & Lechner, 2019). Books in the childhood home can also be considered representative of family scholarly cultures that promote greater educational attainment (Evans et al., 2010). Different indicators of cSES may then influence later-life cognition through different pathways of varying influence.

Of the studies that have tested mediators of the relationships between cSES and later-life cognition, few analyzed specific components of their cSES indicator, fewer with a theory-driven rationale for exploring these components. Two focused on parental indicators of education, occupation, and income (Greenfield & Moorman, 2019; Lyu & Burr, 2016). The other only examined overcrowding in the childhood home, thus no comparative conclusions could be drawn (Cermakova et al., 2018).

## Study Objective

The aim of this study was to estimate the direct effects of a cSES composite (Aartsen et al., 2019; Wahrendorf & Blane, 2015) as well as its individual components on mid to later-life memory performance. Higher levels of education and occupation generally carry more cognitive complexity along with social status; thus, we also explore specific pathways through these potential mediators. We hypothesized that few books in the childhood home would reflect scholarly cultures and have mainly indirect effects on mid to later-life memory performance through facilitating success with educational and occupational pursuits. Indicators of material deprivation (overcrowding and lack of facilities) may have stronger direct affects through biological risks to neurodevelopment, such as increased likelihood of infections and chronic stress. Both parental occupation and the cSES composite reflect intellectual and material resources in the childhood home environment, thus only partial mediation of total effects by adult socioeconomic factors are expected. The exploration of mediated effects through education and occupation also furthers evidence as to whether later interventions in these areas can make up for detrimental effects of early-life socioeconomic disadvantage.

A novel contribution of this study is the use of the g-formula for mediation analyses between cSES and later-life memory performance. Mediation analyses in life course epidemiology can be challenging, as midlife mediators of the relationships between early-life exposures and later-life health outcomes can be confounded by other life course events that are also influenced by the early-life exposure, which is a source of bias. The g-formula is an advanced method that is able to handle these exposure-induced mediator-outcome confounders (EIMOCs), to improve internal validity of life course mediation analyses (Mansournia et al., 2017; VanderWeele, 2016).

## Method

### Sample

Data were from the publicly available English Longitudinal Study of Aging (ELSA), a cohort study of adults aged 50 years and older living in England (Steptoe et al., 2013). The initial ELSA cohort is representative of the English population aged ≥50 years when compared to national census data (Steptoe et al., 2013). The data collection procedures in ELSA were approved by the National Research and Ethics Committee in the U.K.

**Figure 1.**
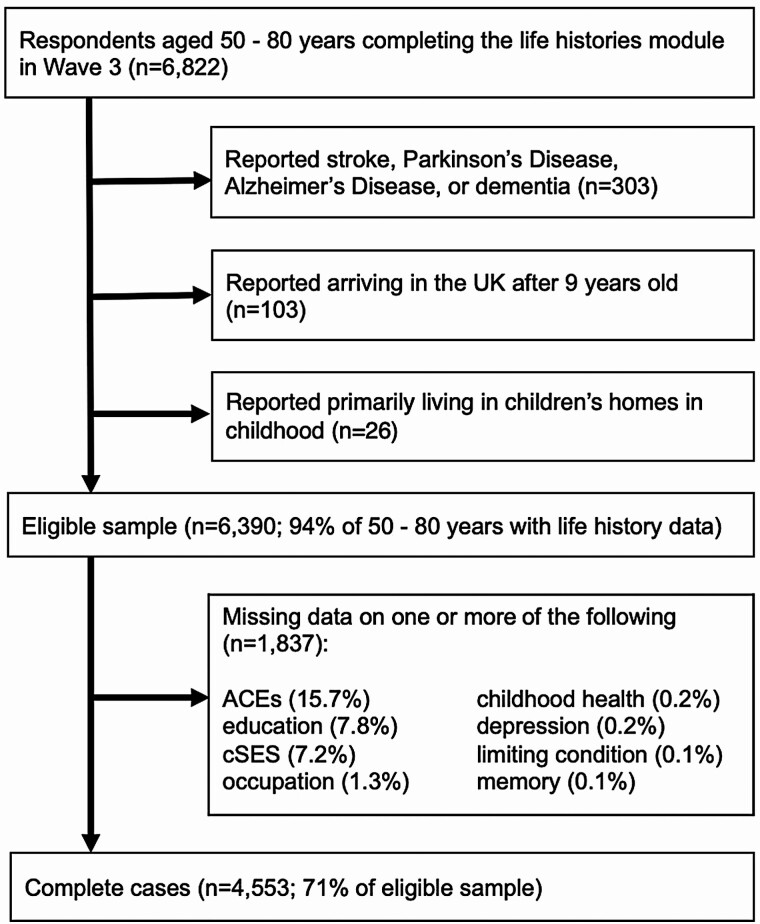
Flowchart of sample inclusion criteria, English Longitudinal Study of Aging, 2006/2007. ACE = adverse childhood experience; cSES = childhood socioeconomic status; UK = United Kingdom.

Participants eligible for the present analysis were aged 50–80 years and completed the life histories module in Wave 3 (2006/2007; *n* = 6,822). All data come from this wave. We used 80 years as an upper limit to minimize bias from differential survival by socioeconomic background. Further exclusion criteria are detailed in Figure 1. Of the eligible sample, there were 4,553 complete cases used in the main analyses. The noncomplete cases were no different than the complete cases in terms of childhood disadvantage and age, though they had slightly lower memory scores (−0.3 points; *p* < .001).

### Mediation and Causal Assumptions

**Figure 2.**
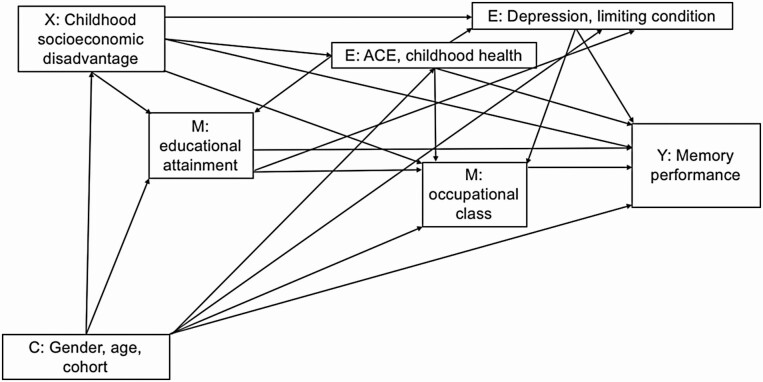
Directed Acyclic Graph reflecting assumed relationships between childhood socioeconomic disadvantage, mediators, and memory performance in older adults. X = exposure; C = baseline confounder; M = mediator; E = exposure-induced mediator-outcome confounder; Y = outcome; ACE = adverse childhood experience.

All mediation methods have assumptions about confounding, including no exposure-mediator confounding, no exposure-outcome confounding, and no mediator-outcome confounding (VanderWeele, 2016). In addition, exposures cannot affect mediator-outcome confounders (VanderWeele, 2016). This final assumption about EIMOCs can very rarely be met by traditional mediation methods, particularly when exposures, mediators, and outcomes are far apart in time (VanderWeele, 2016). Figure 2 further discloses our assumptions about the causal ordering and relationships between variables in this study.

### Exposure: cSES and its Components

cSES was constructed along similar lines to Aartsen et al. (2019) and Wahrendorf and Blane (2015). In Wave 3 of ELSA, life histories were asked with questions about the home and family environments around 10 years of age. Four dichotomous variables reflecting childhood disadvantage around age 10 were constructed: (a) lower-skilled occupation of the family breadwinner; (b) having few books in the home; (c) overcrowding in the home; and (d) having no indoor toilet, no fixed bath, no central heating, and no hot and cold running water supply to the home. The occupation of the family breadwinner was coded based on the International Labor Organization’s definition of skill level (ILOSTAT, n.d.). Managers, senior officials, business owners, professionals, and technicians were coded as highly skilled (skill levels three and four). Armed forces were coded as missing (*n* = 164) due to a lack of clarity about the nature of the work for those employed in this domain as there are a variety of positions in the armed forces (International Labour Office, 2012). All other categories were coded as the lower-skilled group (skill levels one and two). For number of books in childhood, participants were asked if they had none or very few books (0–10 books), enough to fill one shelf (11–25 books), enough to fill one bookshelf (26–100 books), enough to fill two bookshelves (101–200 books), or enough to fill three or more bookshelves (200+ books). Those that responded as having none or very few books were coded as having few books in childhood. Overcrowding was constructed by dividing the number of people living in the home by the number of bedrooms. The number of people per bedroom was considered overcrowded if there were more than two people per bedroom. Our composite variable differs from Aartsen et al. (2019) and Wahrendorf and Blane (2015) in that SHARE asks about the number of rooms versus the number of bedrooms in the childhood home, so they both defined overcrowding as more than 1 person/room, while we used more than 2 persons/bedroom to define overcrowding.

### Mediators: Educational and Occupational Attainment

We considered two mediators that reflect adult socioeconomic status: educational attainment and occupational class. Educational attainment had six ordinal categories based on the U.K. education system: no qualifications, National Vocational Qualifications (NVQ) level 1, NVQ level 2, NVQ level 3, higher education below a degree, and degree-level education. “Foreign qualifications,” “Don’t know,” and “Not asked” were coded as missing.

The respondent’s own occupational class was based on the U.K. National Statistics Socioeconomic Classification (NS-SEC) system, which groups people based on working conditions, relations, and rewards (Galobardes et al., 2006b). We used the three-level schema as only this classification can be treated as having a hierarchical order (Galobardes et al., 2006b). These occupational levels are routine or manual, intermediate, and managerial or professional. Responses of “Not applicable” and “Other” were coded as missing.

### Outcome: Memory Performance

We summed the scores from two episodic memory tasks that involved immediate and delayed recall trials of a 10-word list for a total of 20 points. Responses of “Not applicable” and “Refusal” were coded as missing, while “Don’t know” responses were coded as a zero for each task. The observed composite memory scores ranged from 0 to 20 points, and it was approximately normally distributed over the eligible ELSA sample (kurtosis of 3.2; skewness of −0.3). We selected memory as the cognitive domain of interest because stable declines over adulthood in memory characterize normal cognitive aging, unlike vocabulary and knowledge tasks that show minimal changes until late in the aging process (Hedden & Gabrieli, 2004; Lövdén et al., 2020).

### Confounders

Important confounders in our study were age (continuous, centered at its mean), age-squared (continuous, centered at its mean), gender (man; woman), and birth cohort. Age was squared to account for its possible nonlinear relationships with memory function (Aartsen et al., 2019). Birth cohorts were categorized based on major periods in history that may have differential affects on children’s health and development over the life course, such as war or economic depressions (Hale, 2017; Kesternich et al., 2014). Those born before 1929 made up the first cohort, those born during the Great Depression up until World War II made up the second cohort (1929–1939), those born during World War II made up the third cohort (1939–1945), and the last cohort were the postwar babies (from 1946 onwards). We did not consider these confounders to be EIMOCs.

### EIMOCs

Poor health in childhood and adverse childhood experiences (ACEs) were considered EIMOCs for both mediators. Those who reported having missed school for more than one month due to their health as a child and who reported that their general health in childhood was fair, poor, or varied a great deal were coded as having poor health in childhood. ACEs have also been attributed to slightly weaker memory function in later life (O’Shea et al., 2021). We constructed a binary variable capturing the ACEs included in previous research by O’Shea et al. (2021). Our variable was coded as no events versus any of the following events in the first 16 years of life: a violent attack, a sexual assault, physical abuse by parents, frequent fighting between parents, parents with drinking, drugs, or mental health problems, being separated from the mother for more than 6 months, having ever lived with foster parents or in a children’s home, and/or having lived primarily in a single-mother household.

Chronic depression and long-standing, limiting health conditions may likewise be affected by cSES, impact occupational attainment, and contribute to late-life cognitive outcomes (Frisoni et al., 2000; Paterniti et al., 2002). In ELSA, current depressive symptoms and long-standing, limiting health conditions were self-reported in the same survey period as cognitive testing, with limited possibility to make inferences about disease duration and causal ordering. A long-standing illness suggests a lengthy disease course, but we were unable to determine if the condition limited occupational attainment in adulthood. Likewise, current depression does not necessarily reflect the history of depressive symptoms, though research suggests that over half of depressed individuals in mid to late-adulthood experience persistent depression, and that depression in relatively healthy middle-aged adults is more likely related to chronicity than to other functional or physical health-related sequalae (Gallagher et al., 2013). The lack of temporal ordering to these variables may limit their validity as EIMOCs. It is possible that they act as mediators on the paths from socioeconomic traits in adulthood to memory performance, but an exploration of adult health variables as mediators is beyond the scope of this study.

### Analytical Plan

Descriptive statistics covered observed values from complete cases in the main analysis. Percentages were reported for categorical variables, with means and standard deviations for continuous variables.

We used the g-formula for our mediation analyses. The g-formula is particularly useful for mediation analyses due to its ability to account for EIMOCs, which pose difficulties for parsing out direct and indirect effects due to overadjustment bias and collider bias (Mansournia et al., 2017). Estimates are computed using counterfactuals with three steps: (a) model the observed data; (b) simulate postbaseline mediators, EIMOCs, and outcomes under different exposure regimes using a Monte Carlo simulation procedure; (c) compute effect sizes with Marginal Structural Models (Daniel et al., 2011; Mansournia et al., 2017; Wang & Arah, 2015). Assuming a binary exposure X, natural direct effects (NDEs) are estimated by the difference in the expected outcome when a person is exposed, but the mediator is set to the value it would have been if the person was unexposed―a counterfactual outcome rather than an observed one―compared to the expected outcome when that person is unexposed (Daniel et al., 2011). NDEs capture pathways that do not pass through the specified mediators by preventing mediators from varying over values of the exposure. Natural indirect effects (NIE) capture pathways that pass through the specified mediators. Again assuming a binary exposure, the NIE is estimated by the difference in the expected outcome when the mediator is set to equal the value it takes when X equals 1 compared to the value it takes when X equals 0, while holding X constant at 1 (Daniel et al., 2011). Specific models for all postbaseline variables (mediators, EIMOCs, and outcome) are detailed in Supplementary Table 1 and are consistent with the Directed Acyclic Graph shown in Figure 2.

We modeled the two mediators sequentially, consistent with recommendations in the literature (VanderWeele & Vansteelandt, 2014). First, we modeled memory performance with education as the sole mediator (Model 1). Second, we modeled memory performance with education and occupation together to estimate the added contribution of occupation to indirect effects on memory performance (Model 2). Finally, we modeled memory performance with education and occupation together as mediators, but also added controls for adult health variables (Model 3). We presented outcomes with and without these adult health variables as their status as EIMOCs is tenuous as previously noted. Ninety-five percent confidence intervals were derived from 1,000 bootstrap replications. To analyze the composite indicator of cSES, we specified a mediation analysis and a categorical exposure variable as options when using the *gformula* package for Stata (Daniel et al., 2011). We also grouped those with three and four disadvantages together and considered this group to be the most disadvantaged socioeconomically in childhood as only 42 respondents (<1% of the sample) had all four cSES disadvantages.

To evaluate whether there was a particular driver of the effects of cSES on memory, we tested the four components of cSES individually as well. All specifications for the g-formula were the same, except we specified a binary exposure variable for each of the four individual cSES components while keeping the remaining three components in the model as controls.

### Sensitivity Analysis

Since the g-formula computes over complete cases, we multiply imputed values for all variables with more than 1% of observations missing to address partial nonresponse and to verify the consistency of trends. Variables involving imputation included cSES (7.2% missing) or its components, education (7.8%), respondent’s occupation (1.3%), and ACEs (15.7%). Missing values from the following variables were not imputed: childhood health (0.2%), depression (0.2%), limiting condition (0.1%), and memory score (0.1%). The imputed analyses used 6,365 of the 6,390 cases from the eligible sample (>99%). All analyses were performed using Stata version 13.1 (College Station, TX).

## Results

Individuals with the most disadvantaged cSES were, on average, older, had more adult health concerns, were more likely to have experienced ACEs, less likely to have pursued higher education or had managerial or professional occupations, and had lower memory scores than those with fewer cSES disadvantages (Table 1).

**Table 1.** Characteristics of the Sample by cSES, English Longitudinal Study of Aging, 2006/2007

	Most disadvantaged	Disadvantaged	Advantaged	Most Advantaged
	(*n* = 425)	(*n* = 1,144)	(*n* = 2,081)	(*n* = 903)
Characteristic	*n* (%)/mean (*SD*)	*n* (%)/mean (*SD*)	*n* (%)/mean (*SD*)	*n* (%)/mean (*SD*)
Lower-skilled breadwinner	418 (98%)	1,105 (97%)	1,799 (86%)	0 (0%)
Few books	403 (95%)	646 (56%)	101 (5%)	0 (0%)
Overcrowding	382 (90%)	416 (36%)	73 (4%)	0 (0%)
Lack of facilities	114 (27%)	121 (11%)	108 (5%)	0 (0%)
Male	212 (50%)	538 (47%)	970 (47%)	378 (42%)
Cohort				
Pre-1929	37 (9%)	76 (7%)	107 (5%)	35 (4%)
Great depression	151 (36%)	358 (31%)	516 (25%)	217 (24%)
World War II	105 (25%)	255 (22%)	459 (22%)	236 (26%)
Postwar children	132 (31%)	455 (40%)	999 (48%)	415 (46%)
Poor childhood health	23 (5%)	82 (7%)	149 (7%)	48 (5%)
Any ACE	207 (49%)	531 (46%)	831 (40%)	320 (35%)
Education				
No qualifications	243 (57%)	474 (41%)	449 (22%)	77 (9%)
NVQ 1	35 (8%)	79 (7%)	94 (5%)	16 (2%)
NVQ 2	64 (15%)	245 (21%)	505 (24%)	188 (21%)
NVQ 3	14 (3%)	63 (6%)	199 (10%)	95 (11%)
Higher education without a degree	48 (11%)	159 (14%)	403 (19%)	167 (18%)
Higher education with a degree	21 (5%)	124 (11%)	431 (21%)	360 (40%)
Occupation				
Routine and manual	265 (62%)	610 (53%)	752 (36%)	179 (20%)
Intermediate	86 (20%)	269 (24%)	524 (25%)	234 (26%)
Managerial and professional	74 (17%)	265 (23%)	805 (39%)	490 (54%)
Depression	86 (20%)	182 (16%)	219 (11%)	91 (10%)
Limiting condition	158 (37%)	359 (31%)	589 (28%)	198 (22%)
Age in years	65.4 (8.4)	63.9 (8.5)	62.4 (8.3)	62.2 (7.7)
Memory score	9.7 (3.4)	10.2 (3.3)	11.2 (3.3)	11.9 (3.1)

*Notes:* ACE = adverse childhood experiences; cSES = childhood socioeconomic status; NVQ = National Vocational Qualifications; *SD* = standard deviation.

The most advantaged (0 cSES components) had memory scores 1.69 (95% confidence interval [CI]: 1.31, 2.07) points higher than the most disadvantaged (3 or 4 cSES components) after full adjustment (Table 2). Total, direct, and indirect effects monotonically increased over cSES groups from the most disadvantaged to the most advantaged, indicating a social gradient to memory performance in later life. Memory differences between the most disadvantaged (3 or 4 cSES components) and the disadvantaged (2 cSES components) were mostly mediated by educational attainment and occupational class, while roughly one third of memory differences with the most advantaged (0 cSES components) group were mediated through these pathways. Most of the mediated proportions were attributable to education as models that included occupation as a mediator supplied relatively small increases in mediated proportions (Table 2). This suggests that pathways that pass from cSES to occupation to later-life memory performance, without passing through education first, added proportionally little to the mediated effects.

**Table 2.** Total, Direct, and Indirect Effects of Childhood Socioeconomic Status on Memory Performance in Adults Aged 50 and Older, English Longitudinal Study of Aging (*n* = 4,553)

	cSES				
	(reference: most disadvantaged)	TCE	NDE	NIE	
		(95% CI)	(95% CI)	(95% CI)	% Mediated^a^
Model 1: Education	Most advantaged	1.76	1.34	0.42	24%
		(1.38, 2.13)	(0.97, 1.71)	(0.33, 0.50)	
	Advantaged	1.18	0.80	0.38	32%
		(0.84, 1.52)	(0.46, 1.15)	(0.29, 0.46)	
	Disadvantaged	0.42	0.11	0.30	73%
		(0.05, 0.79)	(–0.24, 0.46)	(0.20, 0.41)	
Model 2: Education and occupation	Most advantaged	1.77	1.23	0.54	31%
		(1.39, 2.14)	(0.85, 1.60)	(0.45, 0.63)	
	Advantaged	1.17	0.69	0.48	41%
		(0.81, 1.52)	(0.34, 1.05)	(0.39, 0.56)	
	Disadvantaged	0.43	0.07	0.36	84%
		(0.05, 0.81)	(–0.29, 0.42)	(0.25, 0.47)	
Model 3: Education and occupation	Most advantaged	1.69	1.14	0.55	33%
		(1.31, 2.07)	(0.76, 1.52)	(0.46, 0.64)	
	Advantaged	1.10	0.63	0.47	43%
		(0.76, 1.45)	(0.29, 0.98)	(0.39, 0.56)	
	Disadvantaged	0.38	0.09	0.29	76%
		(0.01, 0.74)	(−0.26, 0.43)	(0.19, 0.38)	

*Notes:* The reference category would be those with three or four of the disadvantaged components. cSES = childhood socioeconomic status; TCE = total causal effect; NDE = natural direct effect; NIE = natural indirect effect; CI = confidence interval.

^a^(NIE/TCE×100).

Model 1: Controls for centered-age, centered-age squared, gender, cohort, childhood health, and adverse childhood experiences.

Model 2: Same controls as Model 1.

Model 3: Same controls as Model 1 along with depression and limiting health conditions.

When testing cSES components individually, the largest difference in memory performance was between those who were disadvantaged in terms of books versus those that were not (Table 3). Based on the 95% CIs, the total effect size of few books was statistically different from that of overcrowding, but not from the total effect size of the breadwinner’s occupational skill level. The indirect effect of few books in the home was also larger and significantly different from overcrowding across the three models, as was the indirect effect of the breadwinner’s occupation from overcrowding in Models 2 and 3. Few books also had the only consistently significant direct effect across the three models. A lack of facilities had no significant total, direct, or indirect effects on memory while controlling for the other three components.

**Table 3.** Total, Direct, and Indirect Effects of cSES Components on Memory Performance in Adults Aged 50 and Older, English Longitudinal Study of Aging (*n* = 4,553)

cSES	Mediators	TCE	NDE	NIE	
Component		(95% CI)	(95% CI)	(95% CI)	% Mediated^a^
Lower-skilled breadwinner	Model 1:	−0.66	−0.23	−0.43	66%
	Education	(−0.87, −0.44)	(−0.44, −0.01)	(−0.51, −0.35)	
	Model 2:	−0.67	−0.19	−0.48	71%
	Education and occupation	(−0.88, −0.46)	(−0.40, +0.02)	(−0.56, −0.40)	
	Model 3:	−0.67	−0.19	−0.48	72%
	Education and occupation	(−0.88, −0.46)	(−0.40, +0.03)	(−0.56, −0.40)	
Few books	Model 1:	−0.87	−0.41	−0.46	53%
	Education	(−1.09, −0.65)	(−0.62, −0.20)	(−0.55, −0.38)	
	Model 2:	−0.89	−0.39	−0.51	57%
	Education and occupation	(−1.12, −0.67)	(−0.61, −0.16)	(−0.60, −0.42)	
	Model 3:	−0.82	−0.34	−0.48	59%
	Education and occupation	(−1.04, −0.60)	(−0.56, −0.12)	(−0.57, −0.39)	
Overcrowding	Model 1:	−0.34	−0.05	−0.29	84%
	Education	(−0.60, −0.09)	(−0.30, +0.19)	(−0.37, −0.21)	
	Model 2:	−0.35	−0.05	−0.31	87%
	Education and occupation	(−0.60, −0.10)	(−0.28, +0.19)	(−0.39, −0.22)	
	Model 3:	−0.31	−0.02	−0.29	94%
	Education and occupation	(−0.56, −0.06)	(−0.25, +0.22)	(−0.37, −0.21)	
Lack of facilities	Model 1:	−0.31	−0.27	−0.04	13%
	Education	(−0.66, +0.04)	(−0.61, +0.07)	(−0.14, +0.06)	
	Model 2:	−0.32	−0.26	−0.05	17%
	Education and occupation	(−0.66, +0.02)	(−0.59, +0.06)	(−0.16, +0.05)	
	Model 3:	−0.35	−0.29	−0.06	17%
	Education and occupation	(−0.70, +0.00)	(−0.63, +0.05)	(−0.16, +0.04)	

*Notes:* The reference category would be those classified as not having the disadvantaged component. cSES = childhood socioeconomic status; TCE = total causal effect; NDE = natural direct effect; NIE = natural indirect effect; CI = confidence interval.

^a^(NIE/TCE×100).

Model 1: Controls for the remaining three cSES components, centered-age, centered-age squared, gender, cohort, childhood health, and adverse childhood experiences.

Model 2: Same controls as Model 1.

Model 3: Same controls as Model 1 along with depression and limiting health conditions.

Analyses with multiple imputations did not reveal any major deviations from the main trends described (Supplementary Table 2), but rather reinforced statistical differences in effect sizes of the components of childhood disadvantage. These analyses further indicated that the total effect of few books was statistically larger than the total effect of the breadwinner’s occupational skill in Models 1 and 2; the total effect of the breadwinner’s occupational skill was also statistically larger than that of overcrowding in Model 2; and few facilities had significant total effects that were significantly smaller than few books across all three models (Supplementary Table 3).

## Discussion

### Key Findings

In this large, population-based study of older English adults, we found a social gradient to the effect of cSES on later-life memory performance and that mediated proportions through education and occupation were the smallest when groups were most polarized in terms of their childhood socioeconomic conditions. We also found that few books in the childhood home appeared to be an important driver of cSES’ effect on memory performance in later life. Books signify a scholarly culture in the home environment, which can support learning and educational attainment regardless of parental education or class (Evans et al., 2010). We also found that the number of books in the childhood home, unlike other components of cSES, had significant direct consequences on later-life memory performance beyond educational and occupational pathways―possibly through fostering a general intellectual habitus. Consistent with our expectations, when the breadwinner’s occupational skill level or few books in the home marked childhood disadvantage, we found a greater magnitude of indirect effects through the respondent’s own educational and occupational attainment compared to markers of housing conditions in childhood. This is coherent with families’ educational and occupational histories having a primacy in shaping the future aspirations their children (Pimlott-Wilson, 2011), with likely consequences on educational and occupational choices later in the life course.

Overcrowding in the childhood home was less important for later-life memory performance in this analysis, while a lack of facilities had little to no effect. One explanation may be that World War II may have made poor housing conditions more commonplace across socioeconomic standings due to the bombing of housing stock, and the suspension of home improvements and redevelopment initiatives (Stewart, 2005). Likewise, what defines a lack of facilities changes over cohorts and locations (Galobardes et al., 2006a), thus this aspect of cSES on its own may be more associated with a specific birth cohort or rural living than with disadvantage. This could explain our null finding with a lack of facilities in the home. Contrary to our expectations, overcrowding seemed to influence later-life memory mainly through indirect pathways. It may be that children in overcrowded homes are more inclined, or expected, to leave school as soon as possible in order to live independently or support their large families through paid work. Furthermore, overcrowding may signal limited possibilities to pursue focused homework, which is both cognitively stimulating and necessary for school success.

In terms of our mediators, occupation offered a limited additional mediating effect on later-life memory over and above that which passes through education first. This finding is in line with the stabilizing of cognitive returns from intellectual pursuits in late adolescence and is consistent with the sensitive period model in life course epidemiology theory (Ben-Shlomo & Kuh, 2002). Occupation may be less critical to the accentuation of cognitive gaps because it does not fall in the sensitive period. These results suggest that there is scope for intervention on factors occurring after childhood, though more so on education levels, for mid to later life memory. However, the impact may be less substantial when children have few books early on, as seen by the significant direct effect of this exposure.

### Comparisons to Existing Literature

Our findings corroborate studies that find poorer cognitive outcomes with lower levels of socioeconomic status indicators from both childhood and adulthood using ELSA data (Almeida-Meza et al., 2021; Jivraj et al., 2020; Lang et al., 2008). Likewise, our study supports previous findings of partially mediated effects between cSES and cognition at older ages through education and/or occupation (Aartsen et al., 2019; Cermakova et al., 2018; Wolfova et al., 2021). All three of these mediation studies used data from the Survey of Health Aging and Retirement in Europe (SHARE), a sister study of ELSA, which has similar life history information for determining cSES. We constructed our cSES indicator in the same way as Aartsen et al. (2019) and found a similar socioeconomic gradient to memory performance in later-life. The two other SHARE studies used only overcrowding and few books in the home to define childhood socioeconomic disadvantage. Cermakova et al. (2018) found that a selection of health and social risk factors―including educational attainment and current employment―together mediated 45% of cognitive differences across cSES levels, with education alone mediating 35%. Wolfova et al. (2021) estimated the mediating effect of education at 30% for men and 31% for women. With the categorical cSES composite in our study, we found mediated proportions in this range depending on which cSES strata was compared to the most disadvantaged group.

The three studies that found full mediation by adult SES factors all used composite scores based on educational and occupational characteristics of the parents (Beck et al., 2018; McElroy et al., 2021; Oi & Haas, 2019). An explanation for the full mediation may lie in their definitions of childhood disadvantage that were built entirely from indicators of parental education and occupation (Beck et al. 2018; McElroy et al., 2021; Oi & Haas, 2019). In our study, parental occupation was very poor on its own for differentiating those considered most disadvantaged from the other cSES strata, except the most advantaged group. This can be explained by a notable shift in the occupational structure of the U.K. in the mid-Twentieth century (Goldthorpe & Payne, 1986), such that lower-skilled positions would have been the norm for the parents of our sample. In turn, occupational patterns consistent with older cohorts makes the sole exposure of parental occupation less optimal for differentiating socioeconomically disadvantaged childhoods from more average childhoods in our sample, and potentially in other samples as well.

Two of the three studies which found full mediation by adult SES factors included general cognitive abilities in adolescence as mediators and concluded that they were the primary mediators of the relationship between cSES and later-life cognitive function (Beck et al., 2018; McElroy et al., 2021). According to Lövdén et al. (2010), these functional abilities reflect a brain’s existing capacity to address the demands placed on it―its flexibility. A brain’s flexibility increases through adaptive anatomical changes when sustained demands outweigh current capacities, such as through increasing levels of schooling (Lövdén et al., 2010). With this understanding, education and cognitive abilities may work interchangeably in the pathways to memory performance. However, education will likely capture the social background characteristics that facilitate higher academic levels (Leist et al., 2021), while cognitive abilities may capture more of the direct effects related to neurodevelopment (Jensen et al., 2017).

### Study Limitations

One important consideration for interpreting results is the potential for recall bias when reporting childhood conditions in mid to late adulthood. Prior research suggests that retrospective accounts of early-life conditions show moderate accuracy when compared to prospective data collected from childhood (Batty et al., 2005; Jivraj et al., 2020). However, recall accuracy may be more limited with childhood circumstances that reflect historical trends, such as with housing facilities (Jivraj et al., 2020). The null effects seen with a lack of facilities in our study may reflect added difficulties in remembering housing condition details if they were relatively normal conditions for a given cohort. In general, however, conclusions are similar with either retrospectively or prospectively collected childhood socioeconomic data, though both under- and overestimated effect sizes are documented (Batty et al., 2005; Jivraj et al., 2020). Likewise, finer details of retrospectively collected data may be more prone to recall errors than dichotomized data reflecting general conditions (Fransson et al., 2008). Thus, the dichotomization of our individual cSES components in line with Aartsen et al. (2019) and Wahrendorf and Blane (2015) should provide a reasonable indication of the effects of childhood socioeconomic disadvantage on the cSES–cognition relationship.

Another limitation of our study includes nonresponse bias. Partial nonresponse bias arising from missing answers to survey questions was dealt with by multiply imputing data in our sensitivity analysis. Nonresponse arising from a refusal to participate and/or sample attrition remains a potential source of bias. We used Wave 3 data, therefore those lost to follow-up from Wave 1 of ELSA would not be represented in our data. Since survey attrition in ELSA, and nonparticipation in general, have been associated with lower levels of socioeconomic status indicators and health issues (Martikainen et al., 2007; Steptoe et al., 2013), we may lack precision with estimates at the lower ends of our socioeconomic indicator distributions (cSES, education, and occupation). Furthermore, if health follows a social gradient and poorer health and lower socioeconomic standing are associated with nonresponse, then responding individuals from lower social strata may represent the healthiest of the group and lead to more conservative effect estimates. Yet, others report that poorer response rates associated with lower socioeconomic strata do not likely bias health inequality outcomes in a serious way (Martikainen et al., 2007).

In a similar vein, the lack of a sufficient sample with all four of the cSES disadvantages limits distinctions between those with three versus four disadvantages. As the most disadvantaged group in our sample was mostly comprised of those with three disadvantages, the memory performance gaps between those with all four cSES disadvantages versus no disadvantages may in fact be even larger. Aartsen et al. (2019) had a larger sample size and could group those with three and four cSES disadvantages separately. They found a significantly different level of performance on delayed recall tests between these two groups (Aartsen et al., 2019).

Finally, the g-formula can present some limitations to causal inference. The g-null paradox is most commonly described, where model misspecifications are inevitable and lead to biased estimates when EIMOCs are present, and the exposure has no effect on the outcome (McGrath et al., 2022). The g-formula is however an advantageous tool for exploring effects in the presence of multiple exposures over time, such as our mediation analysis with childhood disadvantage, adolescent education, and midlife occupation, and when it would be fairly safe to assume the null hypothesis is untrue (Mansournia et al., 2017). Using the g-formula in this instance appears reasonable given the previously described research that suggests there are at least some direct and/or indirect effects of cSES on adult cognition.

## Conclusions

Composite measures of childhood socioeconomic disadvantage remain important tools for capturing the complexity of early-life adversity, though this study demonstrated that certain aspects of disadvantage may be more relevant than others for mid to late-life cognitive functioning. We found the lack of books in the childhood home mattered more than poor housing conditions for memory performance in mid to late adulthood. Promoting literate households and access to books early in the life course should be relevant strategies for cognitive health in later life, though there appears to be scope for modifying the effects of early socioeconomic disadvantage through educational, and possibly occupational, interventions.

## Supplementary Material

gbac075_suppl_Supplementary_Tables

## Data Availability

Data are publicly available from the UK Data Service: https://beta.ukdataservice.ac.uk/datacatalogue/series/series?id=200011
