# Supplementary material for: Childhood Socioeconomic Disadvantage and Pathways to Memory Performance in Mid to Late Adulthood: What Matters Most?
Source: J Gerontol B Psychol Sci Soc Sci. 2022 May 18;77(8):1478–89. doi: 10.1093/geronb/gbac075 (PMC9371451; doi:10.1093/geronb/gbac075)
Supplement: gbac075_suppl_Supplementary_Tables [file gbac075_suppl_supplementary_tables.pdf]

Supplementary Table 1. Models for the Mediators, EIMOCs and Outcome in the G-formula Analysis

Dependent Variable	Model 1: Explanatory Variables	Model 2: Explanatory Variables	Model 3: Explanatory Variables	Command
Educational attainment	cSES, male, cohort, ACEs childhood health,	Same as model 1	Same as model 1	ologit
ACE	cSES, male, cohort,	Same as model 1	Same as model 1	logit
Childhood health	cSES, male, cohort, ACEs	Same as model 1	Same as model 1	logit
Occupational Class		cSES, male, cohort, ACEs, childhood health, educational attainment	Same as model 2 with depression, limiting health condition added	ologit
Depression			cSES, male, age, cohort, ACEs, childhood health, limiting health condition, educational attainment	logit
Limiting health condition			cSES, male, age, cohort, ACEs, childhood health, educational attainment	logit
Memory score	cSES, male, age, age^2, cohort, childhood health, ACEs, educational attainment	Same as model 1 with occupational class added	Same as model 2 with depression, limiting health condition added	regress

ACE: Adverse childhood experience; cSES: childhood socioeconomic status; EIMOC: Exposure-induced mediator-outcome confounder.

Supplementary Table 2. Total, Direct, and Indirect Effects of Childhood Socioeconomic Status on Memory Performance in Adults Aged 50+ Years with Imputed Data, English Longitudinal Study of Ageing (n=6,365)

	**cSES** *(reference: most disadvantaged)*	**TCE (95% CI)**	**NDE (95% CI)**	**NIE (95% CI)**	**% Mediated**
**Model 1: Education**	Most advantaged	1.84 (1.49, 2.19)	1.39 (1.03, 1.74)	0.45 (0.38, 0.53)	25%
	Advantaged	1.26 (0.95, 1.56)	0.83 (0.52, 1.14)	0.43 (0.35, 0.51)	34%
	Disadvantaged	0.44 (0.12, 0.76)	0.12 (–0.18, 0.43)	0.31 (0.22, 0.41)	71%
**Model 2: Education & Occupation**	Most advantaged	1.77 (1.42, 2.11)	1.24 (0.88, 1.59)	0.53 (0.45, 0.61)	30%
	Advantaged	1.23 (0.91, 1.54)	0.72 (0.41, 1.04)	0.50 (0.43, 0.58)	41%
	Disadvantaged	0.41 (0.09, 0.73)	0.09 (–0.21, 0.39)	0.32 (0.22, 0.42)	79%
**Model 3: Education & Occupation**	Most advantaged	1.78 (1.44, 2.13)	1.24 (0.89, 1.58)	0.55 (0.47, 0.63)	31%
	Advantaged	1.20 (0.90, 1.51)	0.72 (0.41, 1.03)	0.48 (0.41, 0.56)	40%
	Disadvantaged	0.47 (0.16, 0.79)	0.16 (–0.14, 0.46)	0.32 (0.22, 0.41)	67%

TCE: Total Causal Effect; NDE: Natural Direct Effect; NIE: Natural Indirect Effect; CI: Confidence Interval.
†(NIE/TCE*100).
Model 1: Controls for centered-age, centered-age squared, gender, cohort, childhood health, adverse childhood experiences.
Model 2: Same controls as model 1.
Model 3: Same controls as model 1 along with depression and limiting health conditions.
Note. The reference category would be those with three or four of the disadvantaged components.

Supplementary Table 3. Total, Direct, and Indirect Effects of the Childhood Disadvantage Subcomponents on Memory Performance in Adults Aged 50+ Years from ELSA with Imputed Data (n=6,365)

cSES Component	Mediators	TCE (95% CI)	NDE (95% CI)	NIE (95% CI)	% Mediated
**Lower-Skilled Breadwinner Occupation**	Model 1: Education	–0.66 (–0.85, –0.48)	–0.23 (–0.42, –0.05)	–0.43 (–0.50, –0.36)	65%
	Model 2: Education & occupation	–0.67 (–0.85, –0.49)	–0.19 (–0.37, –0.00)	–0.48 (–0.56, –0.41)	72%
	Model 3: Education & occupation	–0.60 (–0.79, –0.41)	–0.14 (–0.33, +0.06)	–0.46 (–0.53, –0.39)	77%
**Few Books**	Model 1: Education	–1.08 (–1.29, –0.88)	–0.55 (–0.75, –0.35)	–0.53 (–0.61, –0.45)	49%
	Model 2: Education & occupation	–1.08 (–1.28, –0.87)	–0.53 (–0.73, –0.32)	–0.55 (–0.63, –0.47)	51%
	Model 3: Education & occupation	–0.98 (–1.18, –0.78)	–0.47 (–0.66, –0.28)	–0.51 (–0.59, –0.43)	52%
**Over-crowding**	Model 1: Education	–0.30 (–0.52, –0.08)	–0.00 (–0.21, +0.21)	–0.30 (–0.37, –0.22)	99%
	Model 2: Education & occupation	–0.27 (–0.48, –0.06)	0.06 (–0.15, +0.27)	–0.33 (–0.41, –0.26)	123%
	Model 3: Education & occupation	–0.25 (–0.47, –0.03)	0.06 (–0.16, +0.27)	–0.31 (–0.38, –0.24)	123%

**Lack of Facilities**	Model 1:	–0.36	–0.27	–0.09	25%
	Education	(–0.66, –0.05)	(–0.56, +0.03)	(–0.18, +0.00)	
	Model 2:	–0.36	–0.28	–0.08	23%
	Education & occupation	(–0.67, –0.06)	(–0.58, +0.01)	(–0.18, +0.01)	
	Model 3:	–0.34	–0.26	–0.08	24%
	Education & occupation	(–0.63, –0.04)	(–0.54, +0.03)	(–0.17, +0.01)	

TCE: Total Causal Effect; NDE: Natural Direct Effect; NIE: Natural Indirect Effect; CI: Confidence Interval.
†(NIE/TCE*100).
Model 1: Controls for the remaining three cSES components, centered-age, centered-age squared, gender, cohort, childhood health, adverse childhood experiences.
Model 2: Same controls as model 1.
Model 3: Same controls as model 1 along with depression and limiting health conditions.
Note. The reference category would be those classified as not having the disadvantaged component.
